# Elevated Facial Behavior Variability During Emotions Contributes to Better Functional Communication in Dyslexia

**DOI:** 10.1007/s10919-025-00490-3

**Published:** 2025-07-30

**Authors:** Amie Wallman-Jones, Eleanor R. Palser, Fate Noohi, Belinda Y. Zhang, Christina R. Veziris, Amanda K. Gerenza, Alexis I. Martinez-Arroyo, Marni Shabash, Ashlin R. K. Roy, Sarah R. Holley, Maria Luisa Gorno-Tempini, Virginia E. Sturm

**Affiliations:** 1 Department of Neurology, University of California, San Francisco, San Francisco, USA; 2 Department of Psychology, Palo Alto University, Palo Alto, USA; 3 Department of Psychology, San Francisco State University, San Francisco, USA

**Keywords:** Neurodevelopment, Learning differences, Adolescence, Nonverbal communication

## Abstract

Dyslexia is a neurodevelopmental condition characterized by reading difficulties, yet there is growing evidence for coinciding social and emotional strengths. In our previous work, we found children with dyslexia displayed greater emotional facial behavior to affective stimuli than their well-reading peers, an enhancement that related to better social skills. Traditional measures provide static “snapshots” of emotional facial behavior but overlook important dynamic information about the face’s movements that may confer interpersonal advantages. Here, we examined whether variability in emotional facial behavior was heightened in children with dyslexia and associated with social communication benefits. We coded the second-by-second intensities of ten emotional facial behaviors in 54 children (ages 7–14) with (*n* = 33) and without (*n* = 21) dyslexia while they watched five emotion-inducing film clips. For each trial, we calculated two facial behavior variability scores: a within-emotion variability score (the second-by-second intensity changes within each category of emotional behavior) and a between-emotions variability score (the total number of changes between categories of emotional behavior). Parents also reported on children’s real-world communication skills. Linear mixed-effects models (controlling for age, sex, and total facial behavior) revealed that children with dyslexia had higher within-emotion facial behavior variability but not higher between-emotions facial behavior variability than those without dyslexia. Across the sample, greater total within-emotion facial behavior variability correlated with higher parent-reported functional communication—the ability to express ideas in ways that others can easily understand. These findings suggest nuanced emotional facial behavior dynamics contribute to social strengths in dyslexia.

**Supplementary Information:**

The online version contains supplementary material available at 10.1007/s10919-025-00490-3.

## Introduction

Dyslexia is a common neurodevelopmental condition characterized by reading challenges that arise irrespective of general intelligence, effort, and education (Shaywitz, 1998; Silani et al., 2005). Although phenotypically heterogeneous, people with dyslexia often have trouble segmenting words into smaller phonological sound units and associating these sound units with written letters and words (Bradley & Bryant, 1983; Frith, 1999; Lyon et al., 2003; O’Brien et al., 2012; Shaywitz, 1998). These reading challenges present a barrier to literary-based education and make it difficult for individuals with dyslexia to meet certain academic demands (Knight, 2021).

Beyond reading difficulties, differences in social behavior and emotions are also common in dyslexia. Some children with dyslexia struggle in the school environment (Brimo et al., 2021; Parhiala et al., 2015; Russell et al., 2015) and exhibit difficulties with language in social settings (Cardillo et al., 2018) as well as experiences of anxiety and depression (Carroll & Iles, 2006). Other children with dyslexia, however, display interpersonal strengths and are highly sociable, empathic, and well-liked (Vail, 1990; Badian, 1986). Differences between studies may reflect the presence of co-occurring conditions (e.g., attention-deficit hyperactivity disorder; Brimo et al., 2021), the precise skill being measured (e.g., linguistic or non-linguistic abilities; Cardillo et al., 2018), and the relationship of the rater to the child (e.g., teacher or parent; Russell et al., 2015). Although this research points to the involvement of emotion systems in dyslexia, few studies have assessed emotions in this population. As short-lived, multifaceted events, emotions guide behavior and influence cognition (Levenson, 2003). Emotional reactivity—the coordinated changes in facial behavior, autonomic nervous system activity, and experience that unfold during emotions—can be measured in a laboratory setting (Levenson et al., 2008) and reveal areas of weakness and strength. In our previous laboratory-based studies, we found children with dyslexia displayed greater autonomic nervous system reactivity than children without dyslexia while watching emotionally evocative and empathy-inducing film clips (Palser et al., 2021; Sturm et al., 2021). Our research has also indicated that children with dyslexia have higher resting respiratory sinus arrhythmia (Palser et al., 2021), a measure of parasympathetic nervous system activity that is associated with numerous social and emotional advantages (Beauchaine et al., 2013). Taken together, these studies suggest that differences in behavioral and physiological responding may contribute to interpersonal strengths in some children with dyslexia.

When considering why emotions are crucial for adaptive social behavior, perhaps nothing is more important than the face. Compared to other species, humans have a rich vocabulary of nonverbal cues and are capable of a wide range of nuanced facial expressions (Dobson, 2012; Elfenbein, 2013; Morecroft et al., 2010). During human emotions, the facial muscles move into prototypical configurations that communicate important information to others about our feelings, personalities, and intentions (Cordaro et al., 2020; Cowen & Keltner, 2019). To quantify emotional facial behavior, researchers can code the presence and intensity of various facial movements on a continuous basis (Sturm et al., 2006, 2008, 2015) and assign them to *a priori* emotion categories (e.g., disgust, sadness, amusement, embarrassment, etc.) that have been determined by previous studies (Gross & Levenson, 1995). Intensity scores, which capture the magnitude of facial movement or muscle contraction (Ekman & Friesen, 1978), may be recorded and later summed or averaged to compute measures of total facial behavior. As more intense facial behaviors are less ambiguous and, thus, more informative than subtle movements (Garcia & Tully, 2020), there are certain social advantages to being more expressive. Displaying less emotional facial behavior in social interactions limits communication and inhibits relationship formation because people cannot easily understand each other’s point of view (Butler et al., 2003). In contrast, people who are more expressive are perceived as more cooperative than their less expressive counterparts (Schug et al., 2010). We have found similar results in our own work. Our studies revealed that children with dyslexia had greater emotional facial behavior than their well-reading peers (Sturm et al., 2021; Palser et al., 2021) and that those who were more expressive had better parent-reported social skills in everyday life (Sturm et al., 2021).

The total amount of facial behavior that a person displays plays a central role in social interactions, but the temporal dynamics of facial movements are also important for shaping communication (Krumhuber et al., 2013). The facial behaviors that arise during emotions have predictable components as well as flexible elements that vary across people and contexts (Elfenbein et al., 2007; Gendron et al., 2014; Jack et al., 2014; Le Mau et al., 2021). Over the course of an emotion or social interaction, facial behavior may vary in at least two ways. First, the intensity of one kind of emotional facial behavior may change over time (herein, “within-emotion facial behavior variability”), allowing the intensity of a single category of emotional behavior to fluctuate. Second, the category of emotional facial behavior that arises may change over time (herein, “between-emotions facial behavior variability”), enabling different types of emotional behavior to appear and disappear from the face (Krumhuber & Kappas, 2005). Whereas within-emotion facial behavior variability is captured when someone modulates the intensity of their smiling behavior over time, between-emotions facial behavior variability reflects transitions between expressions (e.g., amusement and sadness) over time. Both kinds of variability serve important social functions. One prior study found that people judge others who exhibit greater variability in their smiling intensity as more genuine (Krumhuber & Kappas, 2005), which suggests that within-emotion variability confers meaningful social information (Kappas & Descôteaux, 2015). Expressing a range of emotional facial behaviors may also be important. In a study where people rated the personality traits of computer-generated faces, faces that showed greater between-emotions variability were considered more authentic and trustworthy than those with a more limited expressive range (Slepian & Carr, 2019). In sum, these studies suggest that there may be social benefits to displaying emotional facial behaviors that vary in both intensity and content.

Although our prior studies found that children with dyslexia displayed greater emotional facial behavior than children without dyslexia, they did not assess the dynamic movements of the face. To move beyond measures of total facial behavior, which reflect static “snapshots” of behavior but overlook important information held in the temporal fluctuations of facial muscle movements (Keltner et al., 2019), in the present study we focused on the dynamics of facial behavior in dyslexia. Children with and without dyslexia underwent an assessment of emotional reactivity in which they viewed five emotion-inducing film clips chosen to elicit specific positive and negative emotions. We computed a within-emotion variability score, which quantified fluctuations in intensity within each category of emotional facial behavior, and a between-emotions variability score, which captured changes between different categories of emotional facial behavior. We hypothesized that, above and beyond total facial behavior, children with dyslexia would display greater facial behavior variability than those without dyslexia. As people with more variable facial behavior may have a wider repertoire of facial movements and greater flexibility in how they express their thoughts and feelings, we also expected that greater facial behavior variability would relate to better interpersonal communication.

## Materials and Methods

### Participants

Fifty-four children (33 with dyslexia and 21 without dyslexia) were included in the present study. All participants were fluent English speakers between the ages of 7 and 14 who were recruited through the University of California, San Francisco (UCSF) Dyslexia Center. The study protocol was approved by the UCSF Human Research Protection Program. Before completing the study, participants provided verbal assent, and their guardians provided written informed consent. Participants’ guardians reported on participants’ race, household income, and current medication use.

Children with dyslexia were recruited from local or specialized schools for students with dyslexia. For inclusion in the dyslexia cohort, children were required to have a prior and current diagnosis of dyslexia from a licensed psychologist at the time of the study. Participants with dyslexia underwent a comprehensive multidisciplinary evaluation that included a clinical interview, neurological examination, neuropsychological assessment, and language testing. Single-word reading was assessed with Letter-Word Identification and Word Attack, untimed measures from the Woodcock-Johnson IV (Schrank et al., 2014), as well as the timed Test of One-Word Reading Efficiency-Version – Second edition (TOWRE-2; Torgesen et al., 2012). Paragraph reading was assessed using the Gray Oral Reading Ability Test – Fifth Edition (GORT-5; Wiederholt & Bryant, 2012). Testing confirmed that all participants with dyslexia had at least one low reading score (≤ 25th percentile). This more liberal cut-off for reading scores was used because most of the children had received extensive remediation at their schools. Children without dyslexia were recruited from local schools and completed an abbreviated assessment of cognition and reading; they had no reading concerns and scored above the 25th percentile on the TOWRE-2 subtests.

All participants scored above the 9th percentile on Matrix Reasoning, a test of nonverbal reasoning from the Wechsler Abbreviated Scale of Intelligence (WASI; Wechsler, 1999), to confirm their intellectual abilities were not in the impaired range. Children were excluded if they had a history of acquired brain injury, known genetic condition that impacts cognition and development, or other co-occurring neurodevelopmental or psychiatric disorder. Sixteen participants with co-occurring attention-deficit hyperactivity disorder were excluded from a larger sample of children with dyslexia.

### Laboratory Assessment of Emotion

#### Procedure

Participants completed a laboratory assessment of emotion at the UCSF Center for Psychophysiology and Behavior. After being seated in a comfortable chair in a well-lit room, participants were oriented to the testing session and informed that they would be videotaped. Their behavior was then recorded continuously with a semi-obscured, remotely controlled video camera. All stimuli were presented on a 21.5-inch computer monitor placed 4.25 feet in front of the participants. Instructions were presented in both visual and audio formats. Participants completed a battery of tasks designed to assess emotional reactivity, empathy, and emotion regulation, but only the emotional reactivity task was included in the present study.

#### Emotional Reactivity Task

Participants watched five film clips that were selected to elicit specific emotions. At the beginning of the task, participants were presented with the following instructions, “Now you will watch some movies. After each movie, we will ask you some questions. We want to know how YOU feel while watching the movie. If you find the videos too upsetting, please close your eyes. Before each movie, you will see an ‘X’ on the screen. Please relax and try to clear your mind when you see an ‘X’ on the screen. Let’s begin. Watch the ‘X’ please.”

Each trial began with a 60-s resting baseline period in which participants watched a black “X” on a white computer screen. They then viewed an approximately 90-s film clip that elicited a specific positive (i.e., awe, amusement, or nurturant love) or negative (i.e., sadness or disgust) emotion. Participants viewed the film clips in the same order (i.e., awe, sadness, amusement, disgust, and nurturant love). The awe film clip was from either *Lord of the Rings* or *Planet Earth* and showed landscapes and vistas; the sad film clip was from *21 g* and showed a woman crying after receiving bad news about her family; the nurturant love film clip was from *Babies* and showed babies crawling and playing with animals; the disgust film clip showed a human ear being cleaned; and the amusement film clip showed a baby laughing while watching someone ripping up paper. Pilot testing in an independent sample of healthy children indicated that these film clips elicited the target emotions.

After each film, participants responded to a question about the content of the film clip to ensure that they had been paying attention. Participants were then asked if they had seen the film from which the clip was taken, which provided a measure of their prior familiarity with the stimuli. Finally, they rated the extent to which they felt afraid, happy/amused, angry, awe/amazement, disgusted, embarrassed, excited/enthusiastic, love/affection, proud, sad, or surprised while watching each film clip. They were asked, “Did you feel ______ while watching the movie?” and were given the following choices: “no,” “a little,” or “a lot.”

### Laboratory Measures

#### Facial Behavior

Video recordings of participants’ facial behavior were rated by trained coders (Ekman P & Friesen W, 1978) using Noldus version 13.0 software (Noldus Technologies, Leesburg, VA). Coders were blind to the study goals and hypotheses, participant group membership, and did not interact with the participants before, during, or after data collection. Participants’ facial behavior while watching each 30-second film clip was coded on a second-by-second basis using a modified version of the Emotional Expressive Behavior coding system (Gross & Levenson, 1995). Coders rated the following categories of emotional facial behavior: interest, concern, anger, sadness, disgust, fear, contempt, happiness/amusement, surprise, and embarrassment. All behaviors were rated on a three-point intensity scale: 1 (*slight but noticeable*), 2 (*moderate*), or 3 (*strong*). When none of these behaviors was present, facial behavior was coded as neutral. As codes were mutually exclusive, blends of emotional facial behavior were not permitted, and the most dominant emotion was coded. 28% of the videos were coded by multiple coders. Inter-observer agreement was quantified with coefficient kappa, which is the proportion of agreement above that expected to occur by chance (Cohen, 1960). Our coders achieved a Cohen’s kappa value of 0.81, indicating excellent reliability (Fleiss, 1981).

To assess whether participants displayed the expected facial behaviors for each trial, we summed the intensity scores of each code across the 30 s in each trial. We then averaged scores across trials to compute a measure of total facial behavior, which provided a metric of overall expressivity.

##### Within-Emotion Facial Behavior Variability

**Fig. 1:**
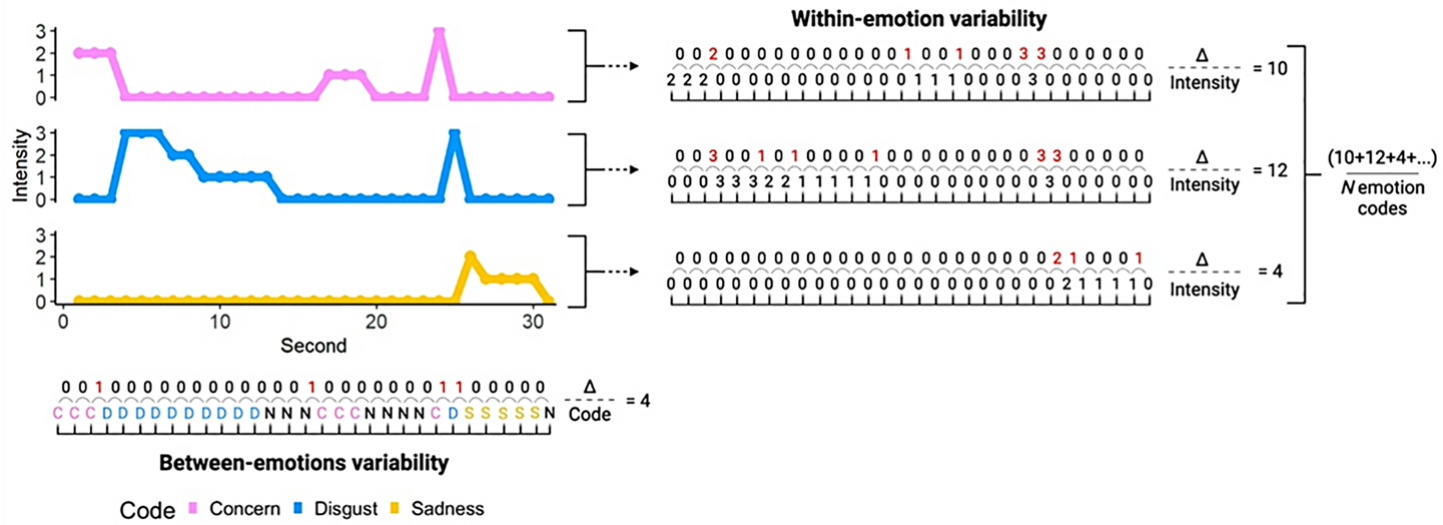
Schematic to illustrate how the within-emotion and between-emotions facial behavior variability scores were calculated from the facial behavior data. *Within-emotion facial behavior variability – the fluctuation in a single category of emotional facial behavior intensity over time;* the second-by-second absolute changes in facial behavior intensity in a single emotion code (e.g., concern) were summed across the 30 s for each emotion code and then averaged across the 10 codes to compute one within-emotion facial variability score per trial. *Between*-*emotions facial behavior variability – the number of changes between emotion codes over time*. The changes between emotion codes (e.g., concern to disgust) were summed across the 30 s to compute one total between-emotions facial variability score for each trial. C = Concern, D = Disgust, N = Neutral, S = Sadness. Created in BioRender. Wallman-Jones, A. (2024) BioRender.com/m94s513

To compute within-emotion facial behavior variability, we quantified the second-by-second changes in intensity for each category of emotional behavior during each trial (Fig. 1). The absolute changes in intensity from one second to the next were summed across the 30 coded seconds using the equation below, where *l*_*1*_ and *l*_*2*_ refer to intensity in adjacent seconds (i.e., *l*_*1*_ refers to facial behavior intensity in second one, and *l*_*2*_ refers to facial behavior intensity in second two):

$$\:Within-emotion\:facial\:behavior\:variability=Abs\:{\sum\:}_{1}^{30}\left({I}_{2}-{I}_{1}\right)$$

**Fig. 2:**
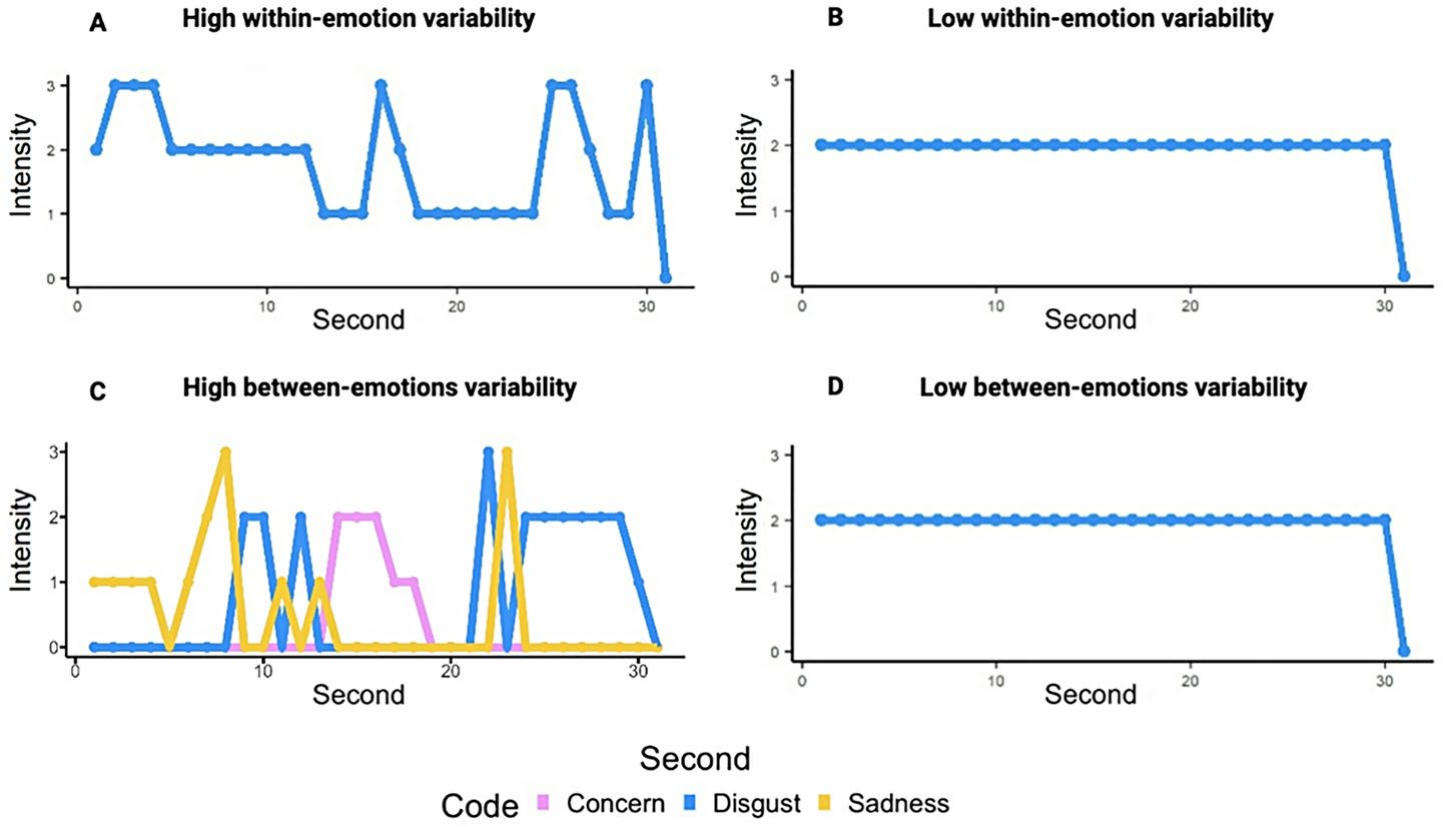
Examples of high and low within- and between-emotions facial behavior variability. **A**) High within-emotion variability; characterized by large second-by-second variability in one emotion code, **B**) Low within-emotion variability; characterized by small second-by-second variability in one emotion code, **C**) High between-emotions variability; characterized by many changes between emotion codes, **D**) Low between-emotions variability; characterized by a small number of changes between emotion codes

These scores were then averaged across codes to produce one within-emotion facial behavior variability score for each of the five trials. Examples of participants with high and low within-emotion facial behavior variability can be seen in Fig. 2A and B.

##### Between-Emotions Facial Behavior Variability

To quantify between-emotions facial behavior variability, we calculated the number of changes between emotion codes during each trial (Fig. 1). For example, if a participant displayed happiness and then disgust, that counted as one change between emotion codes. We did not include changes between an emotion code and neutral facial behavior to ensure that we only measured variability between emotion codes (and not within a code as a participant transitioned between emotional and neutral states). Summing the total number of changes across the coded 30 s produced one between-emotions variability score per trial. Examples of participants with high and low between-emotion facial behavior variability can be seen in Fig. 2C and D.

#### Self-Report Measures

##### Film Clip Content and Familiarity

To ensure that participants had paid attention during the trial, participants were asked a question about the content of the stimuli after viewing each film clip. They were provided with three choices and were asked to identify the correct response. Correct responses were scored 1, and incorrect responses were scored 0. To assess participants’ familiarity with the film clips, they were asked if they had seen each film clip before. Responses were scored (1 = seen before, 0.5 = not sure, 0 = not seen before) and averaged across trials for each participant.

##### Emotional Experience

We coded the responses to the emotional experience questions as 0 (*no*), 1 (*a little*), or 2 (*a lot*). We used these scores to examine whether participants endorsed the target emotional experience in each trial.

### Parent-Reported Measures of Social Communication

A subset of parents (*n* = 24 parents of children with dyslexia, *n* = 19 parents of children without dyslexia) completed the Behavior Assessment System for Children, Second Edition (BASC-2) child and adolescent parent rating forms. The remaining parents declined to complete this measure. The BASC-2 is a standardized, well-validated rating system that assesses a broad range of adaptive and maladaptive behaviors. The child form (ages 6–11) consists of 160 items, and the adolescent form (ages 12–21) consists of 150 items. Item raw scores were summed, and subscale scores were converted into standardized *T* scores. Scores across both forms are standardized within their age group, allowing for comparisons across children and adolescents. Parents were asked to rate each item according to the frequency of the behavior on a 4-point scale, ranging from N (*never*), S (*sometimes*), O (*often*), to A (*almost always*). Raw scores were then summed for each subscale.

Guided by our previous study that found a positive association between total facial behavior and real-world social strengths (Sturm et al., 2021), we focused on two subscales that have relevance for social communication: Functional Communication (the ability to express ideas and communicate in a way others can easily understand) and Social Skills (the skills necessary for interacting successfully with peers and adults in home, school, and community settings). Example items from the Functional Communication subscale include, “Communicates clearly,” “Is able to describe feelings accurately,” and “Likes to talk about his or her day.” Example items from the Social Skills subscale include, “Shows interest in others’ ideas,” “Makes others feel welcome,” and “Encourages others to do their best.”

### Statistical Analyses

Analyses were carried out in R project (R Core Team, 2021) and MATLAB version R2023a (Natick, Massachusetts: The MathWorks Inc.; 2022). Two-tailed tests were used in all analyses.

#### Demographic and Clinical Information

*T*-tests and chi-square tests, or non-parametric Mann-Whitney tests when relevant, were used to assess group differences in age, sex, household income, and cognitive test scores.

#### Emotional Reactivity Task

We used the film content questions to determine whether participants paid attention during each trial. Participants answered 99% of the questions about the film clips correctly, and the remaining incorrect trials (*n* = 3) were excluded from subsequent analyses. We then ran a *t*-test to check for any diagnostic differences in the familiarity of the film clips, which revealed no difference between the groups *t*(57.45) = -0.31, *p* =.761. Finally, we examined the top two emotions that participants displayed on their faces and reported experiencing during each trial to ensure that the film clips induced the intended emotions.

##### Facial Behavior Variability

We ran separate linear mixed-effects models to test for main effects of diagnosis (without dyslexia = 0 and with dyslexia = 1) on within-emotion or between-emotions facial behavior variability. In both models, the variability score for each trial was entered into the model as a repeated measure. Age, sex, total facial behavior (averaged across codes and trials), and a diagnosis-by-trial interaction were entered as fixed effects. Participant and trial were specified as random effects. Given that our prior studies have found heightened total facial behavior in dyslexia (Sturm et al., 2021), we included this measure as a covariate to ensure that any group differences in facial behavior variability were not attributable to overall expressivity. To test whether group differences in within-emotion facial behavior variability were specific to certain emotion codes (rather than certain trials), we also conducted an additional linear mixed-effects model entering the variability score for each emotion code as a repeated measure. Age, sex, total facial behavior (averaged across codes and trials), and a diagnosis-by-emotion code interaction were entered as fixed effects. Participant and trial were specified as random effects. Outliers in the facial behavior variability scores were determined using *Z* scores calculated at the group level, and data were excluded from analyses if they were +/- 3 standard deviations from the mean. Inspection of model residuals via histogram and partial probability plots showed normal distributions.

To assess whether the two facial behavior variability measures reflected a broader construct, we conducted a multiple linear regression that examined the relationship between within-emotion and between-emotions facial behavior variability (controlling for age and sex).

#### Associations Between Facial Behavior Variability and Social Communication

We ran multiple linear regressions to examine whether greater facial behavior variability predicted higher parent-reported scores on the Functional Communication and Social Skills BASC-2 subscales. For these analyses, we computed a single measure of total within-emotion variability and a single measure of total between-emotions facial behavior variability by averaging these scores across trials. Diagnosis (without dyslexia = 0 and with dyslexia = 1), age, sex, and total facial behavior were added as covariates. We also ran exploratory multiple regressions (same covariates) in each diagnostic group separately. We planned to focus these analyses on any facial behavior variability measures that differed between the diagnostic groups in the prior analysis.

## Results

### Demographic and Clinical Information

The sample (reported by 82% of guardians) was predominantly white (67%), followed by multiracial (11%), and Asian or Pacific Islander (4%). The household annual income of the sample (reported by 75% of guardians) ranged from $100,000 to ≥$500,000, which suggests a broad but relatively high socioeconomic status across the sample. Prescription medication use was minimal, with only one participant reporting stimulant use at the time of the laboratory assessment. As expected, the children with dyslexia had lower scores on tests of reading than those without dyslexia yet were similar in nonverbal intelligence. The groups also showed comparable levels of social skills and functional communication, as measured by the BASC-2. See Table 1 for demographic characteristics and descriptive statistics for all measures.

**Table 1:** Participant demographics and cognitive test scores

	Dyslexia	Without dyslexia	*p*
***n***	33	21	
**Age** (Mean (*SD*))	10.10 (1.83)	10.90 (1.85)	0.144
**Sex** (Male/Female)	18/15	10/11	0.828
**Ethnicity**			
Asian or Pacific Islander	1 (3%)	1 (5%)	
White/European American	21 (64%)	15 (71%)	
Multiracial/Multiethnic	4 (12%)	2 (10%)	
Declined to state	7 (21%)	3 (14%)	
**Annual household income**			
$250,000+	18 (55%)	9 (43%)	
$100,000 - $249,000	6 (18%)	10 (48%)	
Declined to state	9 (27%)	2 (9%)	
**WASI: Matrix Reasoning** **(percentile)**	66.80 (24.30)	76.40 (20.00)	0.104
**TOWRE-2: Sight Word** **Effciency Subscale (percentile)**	18.86 (22.41)	60.00 (24.30)	<0.001
**TOWRE-2: Phonemic** **Decoding Effciency** **Subscale (percentile)**	15.21 (16.38)	59.30 (22.70)	<0.001
**Woodcock-Johnson IV**: **Letter-Word** **Identifcation (percentile)**	27.95 (23.28)	NA	NA
**Woodcock-Johnson IV**: **Word Attack (percentile)**	34.39 (24.33)	NA	NA
**GORT-5 Rate (percentile)**	21.07 (15.10)	NA	NA
**GORT-5 Accuracy (percentile)**	12.03 (11.73)	NA	NA
**GORT-5 Fluency (percentile)**	14.58 (11.31)	NA	NA
**BASC-2: Social Skills** **subscale (** ***T*** **-score)**	52.10 (9.72)	57.50 (7.68)	0.784
**BASC-2: Functional Communication** **subscale (** ***T*** **-score)**	51.20 (6.91)	52.80 (8.30)	0.491

T-tests and chi-square tests were used to determine whether there were differences between the groups. Cognitive, academic, and behavioral scores are reported in percentiles; means (M) and standard deviations (SD) are presented unless otherwise noted. Behavior Assessment System for Children, Second Edition (BASC-2), Gray Oral Reading Test Fifth Edition (GORT-5), Test of Word Reading Efficiency – Second edition (TOWRE-2), and Wechsler Abbreviated Scale of Intelligence (WASI). The Woodcock-Johnson tests of achievement and the GORT-5 were not administered in the children without dyslexia in the interest of brevity and retention of participants

### Emotional Reactivity Task

#### Induced Emotions

The measures of facial behavior and emotional experience confirmed that the film clips elicited the intended emotions. The facial behaviors that participants displayed most strongly were happiness/amusement and concern during the awe trial; sadness and concern during the sadness trial; happiness/amusement and embarrassment during the amusement trial; disgust and concern during the disgust trial; and happiness/amusement and interest during the nurturant love trial. The measures of self-reported emotional experience were consistent with the emotions they expressed on their faces. The experiences they reported most strongly were awe/amazement and happiness/amusement during the awe trial; sadness and surprise during the sadness trial; happiness/amusement and excitement/enthusiasm during the amusement trial; disgust and surprise during the disgust trial; and happiness/amusement and love/affection during the nurturant love trial. See Supplementary Tables 1 and 2.

#### Facial Behavior Variability

**Fig. 3:**
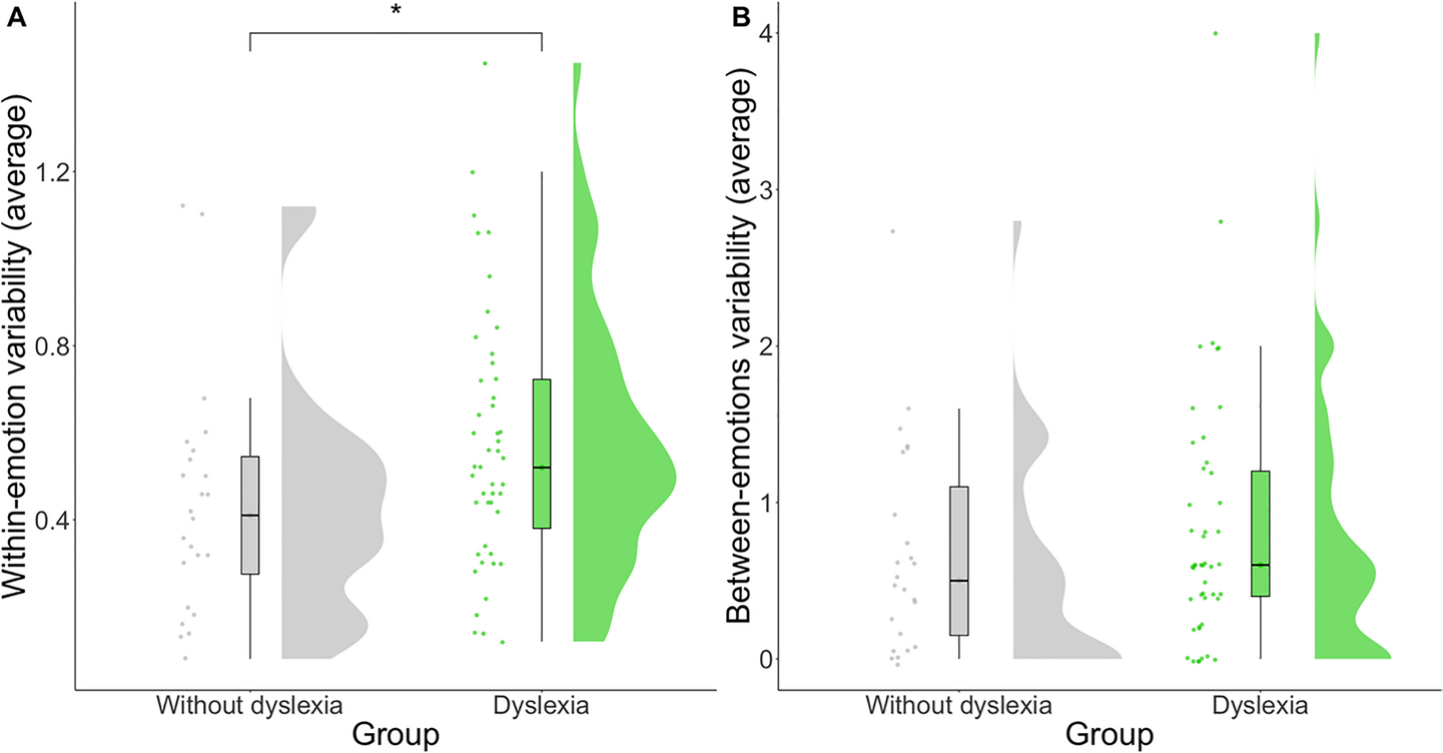
Group effects by diagnosis for (**A**) within-emotion, which considers group differences in the second-by-second change in facial behavior intensity averaged across emotion codes per trial, and (**B**) between-emotions facial behavior variability, which considers group differences in the number of changes between emotion codes per trial. Scores presented here have been averaged across trials for ease of visualization but were entered as repeated measures for each trial in the statistical analyses

Linear mixed-effects models revealed a main effect of diagnosis on within-emotion facial behavior variability, *F*(1,49) = 5.45, *p* =.024 (Fig. 3A), but not between-emotions variability, *F*(1,49) = 0.51, *p* =.477 (Fig. 3B). See Table 2. The diagnosis-by-trial interaction terms for within-emotion, *F*(4,199) = 0.70, *p* =.592, and between-emotions, *F*(4,200) = 0.73, *p* =.573, variability were not significant.

To assess whether the group difference in within-emotion variability was specific to certain emotion codes, we tested a diagnosis-by-emotion code interaction, which was also insignificant, *F*(9,2331) = 1.61, *p* =.106.

Multiple linear regressions revealed a significant correlation between within-emotion and between-emotions facial behavior variability, *B* = 0.33, *t*(49) = 8.52, *p* <.001.

**Table 2:** Within-emotion and between-emotions facial behavior variability scores for each trial. Displayed as means and standard deviations

	Within-emotion variability		Between-emotions variability	
Trial	Dyslexia	Without dyslexia	Dyslexia	Without dyslexia
Awe	0.35 (0.45)	0.38 (0.54)	0.47 (1.08)	0.55 (1.28)
Sadness	0.51 (0.55)	0.36 (0.29)	0.74 (1.26)	0.48 (0.68)
Amusement	0.68 (0.59)	0.65 (0.42)	0.68 (1.19)	1.00 (1.55)
Disgust	0.69 (0.61)	0.46 (0.45)	0.91 (1.47)	1.00 (1.48)
Nurturant love	0.64 (0.62)	0.50 (0.30)	0.94 (1.46)	0.71 (0.85)

### Associations Between Facial Behavior Variability and Social Communication

**Fig. 4:**
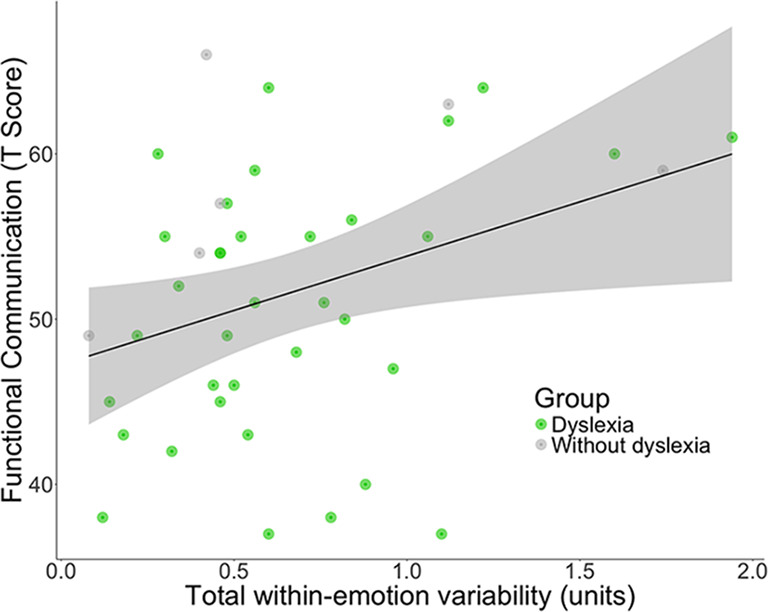
Across the sample, greater total within-emotion facial behavior variability was associated with higher parent-reported functional communication (BASC-2). Total within- emotion variability was calculated as the average second-by-second change in facial behavior intensity for each emotion code, averaged across trials. Functional communication scores are presented as the *T* scores from the BASC-2

Across the sample, multiple linear regressions revealed that greater total within-emotion facial behavior variability (across trials) predicted higher scores in the BASC-2 Functional Communication subscale, *B* = 9.09, *t*(38) = 2.10, *p* =.043 (Fig. 4) but not on the Social Skills subscale, *B* = -6.16, *t*(38) = -1.09, *p* =.281. Although the interaction between diagnosis and within-emotion variability on functional communication was not significant, *B* = -0.02, *t*(37) = -0.62, *p* =.540, exploratory regressions run separately for each diagnostic group revealed a stronger positive relationship between within-emotion variability and functional communication in those with dyslexia, *B* = 9.09, *t*(19) = 2.10, *p* =.061, than in those without dyslexia, *B* = 10.54, *t*(14) = 1.06, *p* =.306. As the between-emotions variability score did not differ between the groups, we did not correlate this measure with the social communication scores.

## Discussion

We found within-emotion facial behavior variability was elevated in children with dyslexia and associated with better parent-reported functional communication. Compared to children without dyslexia, those with dyslexia showed greater second-by-second fluctuations in the intensity of emotional facial behaviors as they unfolded over time. Between-emotions facial behavior variability, a measure that reflected the number of changes between different categories of facial behavior, did not differ between the groups, however. Across the sample, children with greater total within-emotion facial behavior variability had higher functional communication, but not social skills, in everyday life.

During emotions, the movements of the face have a predictable structure but also show dynamic variation over time (Elfenbein, 2013). In most studies, facial behaviors are averaged over prolonged periods of time (from seconds to minutes), an approach that treats emotions as sustained states rather than short-lived responses with their own important temporal signatures (Pasquini et al., 2022). Variability in facial behavior, though often overlooked, adds nuance and subtlety to emotional expressions and broadens the range of communication signals that the face can produce. Here, we leveraged the second-by-second changes in the types and intensities of facial behaviors that children displayed while watching emotion-inducing film clips to quantify two types of facial behavior variability. Even when controlling for total facial behavior across trials, within-emotion facial behavior variability was greater in children with dyslexia than in those without. Between-emotions facial behavior variability, in contrast, did not differ between the groups. We also found that heightened within-emotion facial behavior variability was not specific to any trial or emotion code, instead reflecting a broader pattern across emotions and contexts. Although within and between-emotions variability were correlated, indicating a shared component of emotional variability, the group difference in within-emotion variability suggests that these are not fully interchangeable constructs. These findings indicate that children with dyslexia did not display a greater range of emotions on the face than those without dyslexia but rather that they exhibited greater fluctuations in the intensities of the emotions that they expressed, supporting the idea that their expressions are not necessarily more ambiguous.

Like languages, emotions have their own grammar that guides social interactions (Eibl-Eibesfeldt, 1989). In the present study, we found children with higher within-emotion facial behavior variability had higher parent-rated functional communication. Although the groups did not differ in their average levels of functional communication, this finding suggests that children who display emotional facial behaviors of varying intensities—a tendency that was heightened in dyslexia—are more effective at conveying their ideas in a contextually informed manner. Although we observed no interaction effect between diagnosis and within-emotion facial behavior variability, we found a trend towards a stronger relationship between within-emotion variability and functional communication in those with dyslexia than in those without, suggesting that this relationship may be more pronounced in individuals with learning differences. While more research is needed, higher within-emotion facial behavior may promote the types of nonverbal communication skills that individuals with dyslexia often exhibit.

The present study contributes to a growing body of evidence that people with dyslexia are highly attuned to social and affective cues. In our prior work, we found children with dyslexia exhibited greater total facial behavior and autonomic reactivity in response to emotionally evocative film clips (Sturm et al., 2021). Children with dyslexia also displayed greater facial expressions of concern while watching empathy-inducing film clips while also producing greater resting respiratory sinus arrhythmia (Palser et al., 2021), a parasympathetic measure of heart rate variability. By orienting attention to others and encouraging interpersonal engagement (Porges, 2009), the parasympathetic nervous system encourages prosocial behavior (Sturm et al., 2018), emotion recognition (Quintana et al., 2012), cooperation (Beffara et al., 2016), and empathy (Lischke et al., 2017) in people across the lifespan. Whether elevated parasympathetic nervous system activity in dyslexia fosters variability in the activity of the face, as it does in the activity of the heart, is unknown but worthy of future study.

There are limitations of the current research to consider. First, we quantified facial behavior using a modified version of the Emotional Expressive Behavior coding system, which classifies facial behaviors according to prototypical emotion categories (Gross & Levenson, 1995). While this approach is useful because it reduces complex streams of behavior into mutually exclusive codes, it loses the fine-grained information about the temporal dynamics of specific facial movements. It is possible, therefore, that we did not capture the full range of facial behavior variability with this method, and future studies could elaborate on our results by using more detailed facial coding strategies. Second, dyslexia is a heterogeneous disorder (Shaywitz, 1998) and likely includes multiple behavioral and cognitive phenotypes (O’Brien et al., 2012; Palser et al., 2022) with distinct patterns of facial behavior variability. Thus, it is possible that while some children with dyslexia exhibit these nonverbal strengths, others may not. Additional studies will be needed to understand the emotional and social landscapes of different dyslexia phenotypes and the extent to which our findings apply to children with different types of reading challenges. The generalizability of our findings may also be hindered by the relatively homogenous demographic characteristics of our sample, with many of the participants being white/European American and of higher socioeconomic status. Future studies would benefit from greater ethnic and demographic diversity where cultural differences in emotional expression (i.e., display rules) may influence facial behavior dynamics. Third, we found a positive association between within-emotion facial behavior variability and functional communication, a social strength, but having more dynamic facial behavior may also come with a downside. In our prior study of dyslexia, the children who had more expressive faces not only had better social skills but also had greater symptoms of anxiety and depression (Sturm et al., 2021). Additional research on the role that heightened emotional reactivity, including greater facial behavior variability, plays in affective symptoms will be important for identifying children with dyslexia who might benefit from interventions that help them to manage strong emotions.

The human face conveys rich information about our internal states, and dynamic facial behaviors are critical for flexible social communication. Unlike prior studies that have averaged facial behavior over time, in the present study we examined second-by-second changes in facial movements in children with dyslexia as they viewed a series of emotion-inducing film clips. Heightened within-emotion facial behavior variability characterized the children with dyslexia and was associated with better real-world functional communication. This study emphasizes the need for new analytic approaches that leverage continuous measures of facial behavior over time and adds to the emerging picture of dyslexia as a condition characterized by socioemotional strengths as well as reading weaknesses. Expanded conceptualizations of dyslexia that include nonverbal advantages will be fundamental for improving the treatment, prognosis, and well-being of individuals with learning differences.

## Electronic Supplementary Material

Below is the link to the electronic supplementary material.

Supplementary Material 1

## Data Availability

No datasets were generated or analysed during the current study.
