# Supplementary material for: Elevated Facial Behavior Variability During Emotions Contributes to Better Functional Communication in Dyslexia
Source: J Nonverbal Behav. 2025 Jul 30;49(3):325–43. doi: 10.1007/s10919-025-00490-3 (PMC12408770; doi:10.1007/s10919-025-00490-3)
Supplement: Supplementary file 1 — Supplementary Material 1 [file 10919_2025_490_MOESM1_ESM.docx]

**Supplementary material**

**Table S1**. Total facial behavior for each emotion code in each trial across the whole sample. Displayed as means (*M*) and standard deviations (*SD*).

| **Trial** | **Total facial behavior** | ***M*** | ***SD*** |
| --- | --- | --- | --- |
| **Awe** | Anger | 0.01 | 0.14 |
|  | Concern | 0.06 | 0.29 |
|  | Contempt | 0.02 | 0.18 |
|  | Disgust | 0.00 | 0.09 |
|  | Embarrassment | 0.00 | 0.05 |
|  | Fear | 0.00 | 0.06 |
|  | Happiness/amusement | 0.10 | 0.34 |
|  | Interest | 0.03 | 0.21 |
|  | Sadness | 0.02 | 0.13 |
|  | Surprise | 0.00 | 0.00 |
| **Sadness** | Anger | 0.02 | 0.19 |
|  | Concern | 0.09 | 0.35 |
|  | Contempt | 0.01 | 0.15 |
|  | Disgust | 0.06 | 0.34 |
|  | Embarrassment | 0.01 | 0.18 |
|  | Fear | 0.02 | 0.18 |
|  | Happiness/amusement | 0.08 | 0.30 |
|  | Interest | 0.07 | 0.32 |
|  | Sadness | 0.12 | 0.48 |
|  | Surprise | 0.00 | 0.00 |
| **Amusement** | Anger | 0.00 | 0.05 |
|  | Concern | 0.03 | 0.20 |
|  | Contempt | 0.02 | 0.19 |
|  | Disgust | 0.00 | 0.04 |
|  | Embarrassment | 0.05 | 0.32 |
|  | Fear | 0.02 | 0.17 |
|  | Happiness/amusement | 1.22 | 1.04 |
|  | Interest | 0.01 | 0.13 |
|  | Sadness | 0.01 | 0.14 |
|  | Surprise | 0.00 | 0.00 |
| **Disgust** | Anger | 0.04 | 0.22 |
|  | Concern | 0.14 | 0.46 |
|  | Contempt | 0.00 | 0.05 |
|  | Disgust | 0.55 | 0.97 |
|  | Embarrassment | 0.01 | 0.11 |
|  | Fear | 0.03 | 0.26 |
|  | Happiness/amusement | 0.03 | 0.20 |
|  | Interest | 0.03 | 0.21 |
|  | Sadness | 0.09 | 0.38 |
|  | Surprise | 0.00 | 0.00 |
| **Nurturant love** | Anger | 0.00 | 0.04 |
|  | Concern | 0.05 | 0.27 |
|  | Contempt | 0.02 | 0.19 |
|  | Disgust | 0.02 | 0.21 |
|  | Embarrassment | 0.02 | 0.22 |
|  | Fear | 0.00 | 0.05 |
|  | Happiness/amusement | 0.35 | 0.69 |
|  | Interest | 0.07 | 0.35 |
|  | Sadness | 0.01 | 0.09 |
|  | Surprise | 0.00 | 0.00 |

**Table S2**. Self-reported emotional experience for each emotion code in each trial across the whole sample. Displayed as means (*M*) and standard deviations (*SD*).

| **Trial** | **Self-reported experience** | ***M*** | ***SD*** |
| --- | --- | --- | --- |
| **Awe** | Afraid | 0.00 | 0.00 |
|  | Happy/amused | 0.74 | 0.68 |
|  | Angry | 0.04 | 0.27 |
|  | Awe/amazement | 0.83 | 0.70 |
|  | Disgusted | 0.00 | 0.00 |
|  | Embarrassed | 0.04 | 0.19 |
|  | Excited/enthusiastic | 0.49 | 0.67 |
|  | Love/affection | 0.15 | 0.41 |
|  | Proud | 0.15 | 0.50 |
|  | Sad | 0.08 | 0.27 |
|  | Surprised | 0.30 | 0.54 |
| **Sadness** | Afraid | 0.40 | 0.54 |
|  | Happy/amused | 0.09 | 0.28 |
|  | Angry | 0.11 | 0.37 |
|  | Awe/amazement | 0.00 | 0.00 |
|  | Disgusted | 0.09 | 0.28 |
|  | Embarrassed | 0.02 | 0.15 |
|  | Excited/enthusiastic | 0.00 | 0.00 |
|  | Love/affection | 0.11 | 0.31 |
|  | Proud | 0.00 | 0.00 |
|  | Sad | 1.19 | 0.77 |
|  | Surprised | 0.55 | 0.62 |
| **Amusement** | Afraid | 0.00 | 0.00 |
|  | Happy/amused | 1.40 | 0.89 |
|  | Angry | 0.00 | 0.00 |
|  | Awe/amazement | 0.20 | 0.45 |
|  | Disgusted | 0.00 | 0.00 |
|  | Embarrassed | 0.00 | 0.00 |
|  | Excited/enthusiastic | 1.20 | 0.84 |
|  | Love/affection | 0.40 | 0.55 |
|  | Proud | 0.00 | 0.00 |
|  | Sad | 0.00 | 0.00 |
|  | Surprised | 0.60 | 0.55 |
| **Disgust** | Afraid | 0.22 | 0.46 |
|  | Happy/amused | 0.04 | 0.19 |
|  | Angry | 0.07 | 0.33 |
|  | Awe/amazement | 0.14 | 0.43 |
|  | Disgusted | 1.72 | 0.56 |
|  | Embarrassed | 0.04 | 0.19 |
|  | Excited/enthusiastic | 0.04 | 0.19 |
|  | Love/affection | 0.00 | 0.00 |
|  | Proud | 0.02 | 0.14 |
|  | Sad | 0.04 | 0.19 |
|  | Surprised | 0.74 | 0.78 |
| **Nurturant love** | Afraid | 0.00 | 0.00 |
|  | Happy/amused | 1.13 | 0.82 |
|  | Angry | 0.04 | 0.27 |
|  | Awe/amazement | 0.40 | 0.63 |
|  | Disgusted | 0.00 | 0.00 |
|  | Embarrassed | 0.04 | 0.19 |
|  | Excited/enthusiastic | 0.58 | 0.66 |
|  | Love/affection | 0.69 | 0.79 |
|  | Proud | 0.13 | 0.34 |
|  | Sad | 0.02 | 0.13 |
|  | Surprised | 0.22 | 0.50 |
